# Role and competency model for coordination tasks in municipal health promotion: findings from an integrative review

**DOI:** 10.1186/s12889-025-21689-5

**Published:** 2025-02-21

**Authors:** Patricia Tollmann, Sven Dieterich, Maja Kuchler, Janna Leimann, Laura Lubosch, Vivien Mielenbrink, Pia Rangnow, Stefanie Terhorst, Eike Quilling

**Affiliations:** Department of Health Sciences, Bochum University of Applied Sciences, Gesundheitscampus 6-8, 44801 Bochum, Germany

**Keywords:** Health promotion, Municipal health promotion, Health equity, Competency model, Framework, Coordination, Management, Competencies

## Abstract

**Background:**

Integrated municipal health promotion is becoming increasingly important in promoting health equity. Successful implementation of integrated municipal health promotion processes requires a central coordinator. Therefore, this study investigates which competencies are necessary to successfully manage the extensive coordination tasks. The aim is to present the necessary roles and competencies in a way that can be applied in practice.

**Methods:**

An integrative review was conducted. As a first step, a systematic search was carried out in the databases LIVIVO, Web of Science and via the EBSCOhost platform (including APA PsycInfo, ERIC, MEDLINE, CINAHL, APA PsycArticles, SocINDEX) using various inclusion and exclusion criteria (e.g. English and German language). The steps of systematic data extraction, data evaluation and data analysis were subsequently carried out. In accordance with the integrative review method, the data were then iteratively compared and synthesised, followed by the final step of presentation and integration in the form of a new model.

**Results:**

According to the pre-defined inclusion and exclusion criteria, 12 hits were included. The results identified were combined into a concept for a role and competency model. Central competencies for the coordination of municipal health promotion are e.g., public health expertise, interprofessional competencies as well as coordination, communication, and management competencies.

**Conclusions:**

The role and competency model presents the roles and competencies needed to coordinate integrated municipal health promotion processes and emphasises the need to work intersectorally. The model provides a guide for practical application and can contribute to the selection of qualified staff, professionalisation, training and curriculum development. It can make a quality-assured contribution to the structural development of integrated health promotion at local level. Further research is needed to validate the model in practice.

**Trial registration:**

This integrative review was not registered beforehand.

**Supplementary Information:**

The online version contains supplementary material available at 10.1186/s12889-025-21689-5.

## Background

As early as 1986, the World Health Organization defined the goal of health promotion in the Ottawa Charter as the creation of health-promoting environments to influence the determinants of health so that people are empowered to maintain and improve their health in a self-determined manner [1, 2]. The importance of health promotion is emphasised in the Sustainable Development Goals (SDG’s), particularly SDG 11, which highlights the municipal setting and health promotion actors as pivotal for the creation of cities and communities that are inclusive, safe, resilient, and sustainable [3]. For the implementation of SDG 11, the municipality can, for example, ensure access to affordable housing, safe public transportation, and adequate green spaces and shaded areas. To this purpose, the "Health in All Policies" strategy aims to include health promotion across all sectors and at all levels of policy-making, especially in municipal settings [1, 4–6]. The creation of healthy living environments can only be achieved if sectors other than the health sector are involved and socio-spatial issues are taken into account [7]. Municipalities are systems with sectoral organisations, such as day-care centres, schools, businesses and utilities [8]. They are particularly important for health promotion and offer many opportunities to reach all citizens to promote health equity [8, 9]. One example of an evidence-based approach to municipal health promotion is “Communities That Care”, which is a systemic intervention that integrates existing local structures and organisations and involves them in the entire process via new decision-making and development bodies [10]. By adopting a holistic approach to integration and cooperation, coordinated and cross-sectoral collaboration is becoming increasingly important [5, 11–14]. This requires centrally coordinated municipal health promotion [9, 13].

Quilling et al. [8] defined central tasks of municipal health promotion in an action model, in order to develop a comprehensive strategy that avoids overlapping structures and aims to create health-promoting living environments. Within the process of municipal health promotion, stakeholders from different municipal sectors (e.g., health, planning, social affairs, and politics) must be identified and coordinated. Further tasks consist of project and network management, deriving municipal needs and strategies, evaluation and quality assurance as well as the promotion of participation and communication [8]. Various competencies are required for professionals to accomplish these coordination tasks [8, 15, 16].

Identifying (core) competencies is important for improving the quality and professionalism of health promotion practice, education and research [16]. Competencies provide a basis for professional development, quality assurance, job descriptions and the creation of a more unified workforce [17]. The term 'competencies' should not be interpreted in a one-dimensional way as knowledge, but as a combination of knowledge, skills, abilities and attitudes [16]. A competent workforce with the necessary knowledge, skills and abilities from policy, theory and research can implement health promotion activities to a certain standard [15, 16].

Although there are recognized competency frameworks [18–20], a previous unsystematic literature screening showed that they mostly focus on educational aspects in the public health sector. None of the frameworks present comprehensive competencies needed to coordinate municipal health promotion. Therefore, the aim of this research is to determine the roles and competencies needed to manage municipal coordination tasks as identified by Quilling et al. [8] and to present these competencies in an applicable way for municipal practitioners. The study also aims to examine existing models and frameworks that exist in comparable settings.

Based on the identified research gap that there is no practical orientation to competencies in this context, this research focuses on the following research question: *What roles and competencies are required to successfully manage coordination tasks in municipal health promotion?*

## Methods

For answering the research question, the methodology of an integrative review according to Whittemore & Knafl [21] was chosen to identify roles and competencies for the coordination of municipal health promotion and to present them in a practice-oriented way. Integrative reviews allow the inclusion of various methodologies. They aim to present the current state of evidence, contribute to theory development, and assess the applicability to practice and policy. In contrast to systematic reviews, the integrative review method is less time-consuming, but still offers a more systematic approach than narrative reviews [21, 22].

To develop the integrative review, a systematic literature search was conducted from April to July 2020. The researchers started with the definition of inclusion and exclusion criteria based on the research question. For the selection of publications at title, abstract and full text level, the inclusion or exclusion of publications was decided on the basis of the following categories: types of studies and literature, the phenomenon studied, the setting, the language of the publication, the geographical location and the time period covered by the review and its rationale.

Study types such as reviews, handbooks and different study designs (e.g. Delphi survey, participatory and qualitative studies) were included. Publications were included if they provided overarching information on the roles or competencies needed to manage the coordination of health promotion in a municipal setting. Thus, publications on individual health promotion interventions or on other roles and competencies such as cultural competence were excluded. Only publications written in German and English and with full text available were included. Publications from countries in the Global North were included in order to focus on regions with more comparable socio-economic, developmental and political parameters. Publications published between 2005 and 2020 were included. As there has been an increase in publications on municipal health promotion since 2005, publications older than 15 years at the time of the search were excluded.

The next step was the selection of relevant scientific databases. Social science, medical and health science databases were included in order to include as many intersectoral aspects as possible in answering the research question. This led to the selection of the databases LIVIVO, Web of Science and EBSCOhost (including APA PsycInfo, ERIC, MEDLINE, CINAHL Complete, APA PsycArticles, SocINDEX with Full Text). To develop a suitable search strategy, synonymous terms for competencies in municipal health promotion were tested and defined. Search operators were used in alternating combinations, to obtain as many matching hits as possible. Integrative reviews synthesise diverse literature, including studies that have not yet been indexed or tagged with standardised keywords, so individual indexed search terms were not used. This resulted in the final English- and German-language search string for the systematic search, which was applied to all of the above-mentioned scientific databases with individual adjustments:

- (((kompetenz*[Title] OR rolle*[Title] OR lernziel*[Title] OR fähigkeit*[Title] OR competency*[Title] OR competencies[Title] OR competent[Title] OR role*[Title] OR expertise*[Title] OR ability[Title] OR abilities[Title]) AND (modell*[Title] OR übersicht*[Title] OR model*[Title] OR framework*[Title])) AND (gesundheitsförder*[Title] OR health care[Title] OR health promot*[Title] OR (municipal*[Title] AND health promot*[Title]) OR (local*[Title] AND prevention*[Title]))).

The search strategy was supplemented by an unsystematic online search using the search terms in various combinations in Google scholar and by snowballing. In the next step, all identified studies were screened for eligibility using the pre-defined inclusion and exclusion criteria. In accordance with the PRISMA recommendations [23], the search protocol and the preliminary hit list were reviewed by two researchers and checked for possible inconsistencies in the search strategy. Conflicts regarding the decision to include articles were discussed.

As the next steps in the integrative review method [21], the researchers conducted the steps “data evaluation” and “data analysis”. For this purpose, the relevant data was extracted systematically from the studies and assigned to the respective category according to the research objective (role / competency / model). During the quality appraisal, aspects such as authenticity and information value [21] were evaluated. Authenticity addressed the legitimacy and trustworthiness of the included publications, while informational value assessed whether they provided a relevant basis for the model.

This was followed by iterative data comparison, analysis of existing models and data reduction. According to Toronto and Remington [22], synthesising the results in an integrative review enables a holistic understanding of a given phenomenon. Whittemore and Knafl [21] describe this step as the final phase, “presentation and integration”. In this step, the systematically gained findings were summarised into the concept of a new role and competency model for the coordination of municipal health promotion. In addition to the existing models and frameworks identified in the literature review, the model also draws on additional information from the identified literature.

## Results

**Fig. 1:**
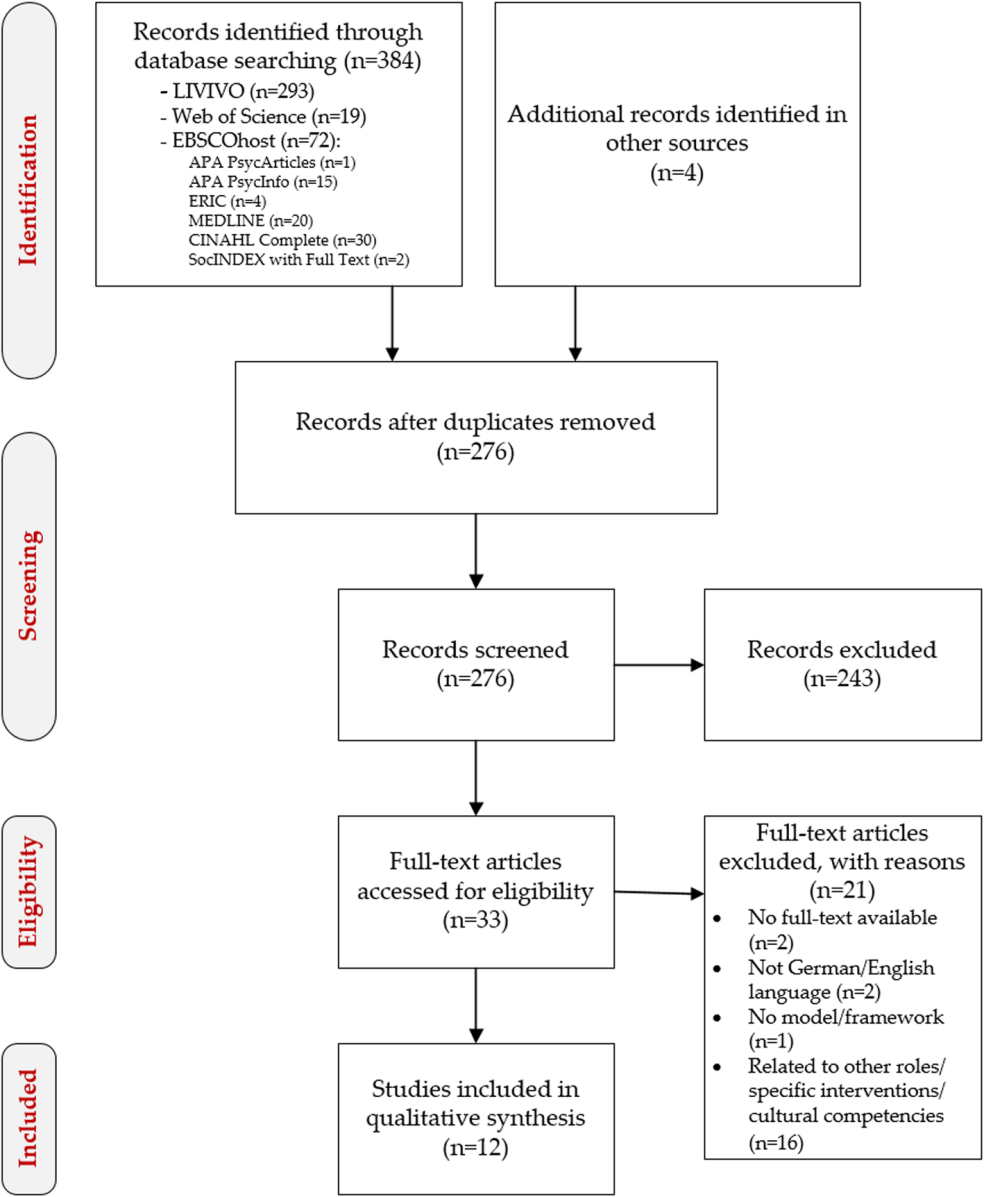
Flowchart of the research and selection process, following Moher et al. [24]

The literature search yielded a total of 318 hits. After removing duplicates and scanning titles, 276 publications remained. The identified abstracts were then systematically analysed for their suitability for further inclusion in the evaluation process. After applying the defined inclusion and exclusion criteria, 33 publications were analysed in full text. Of these, 12 evaluation studies and research reports were selected. Figure 1 shows the results of the search and the inclusion steps based on the PRISMA scheme.

### Results of the literature review

The following table (Table 1) shows which models and frameworks are mentioned in these 12 publications. In total, nine models from the fields of health promotion, medicine and quality assurance are described in detail in the included publications. A detailed summary of findings table (including authors, date, country, title, publication type and methods, summary) is provided as additional file (see Additional file 1, Table S1).

**Table 1:** Brief summary of findings with a focus on presented models and frameworks

**Authors**	**Country of author origin**	**Title**	**Presented frameworks**
Barry et al. (2014) [15]	Germany	Das CompHP-Rahmenkonzept für die Gesundheitsförderung. Kernkompetenzen - professionelle Standards - Akkreditierung	CompHP Core Competencies Framework for Health Promotion
Barry et al. (2012) [25]	Ireland	The CompHP core competencies framework for health promotion in Europe	CompHP Core Competencies Framework for Health Promotion
Batt et al. (2019) [26]	Australia, Canada	The development of competency frameworks in healthcare professions: a scoping review	CanMEDS Framework ACGME Outcomes project entry-level registered nurse practice competencies
Battel-Kirk et al. (2009) [16]	Ireland, United States	A review of the international literature on health promotion competencies: identifying frameworks and core competencies	Several international health promotion competencies frameworks, e.g., the National Health Educator Competencies Update Project (CUP)
Battel-Kirk et al. (2012) [27]	Malta, Netherlands, Italy, Spain, Ireland	Developing a competency-based Pan-European Accreditation Framework for Health Promotion	CompHP Pan-European Accreditation Framework
Dempsey et al. (2011) [17]	Ireland	The CompHP Core Competencies Framework for Health Promotion Handbook	CompHP Core Competencies Framework for Health Promotion
Garman & Scribner (2011) [28]	United States	Leading for Quality in Healthcare: Development and Validation of a Competency Model	Quality Leadership Developmental Competency Model
Glegg & Hoens (2016) [29]	United States	Role Domains of Knowledge Brokering: A Model for the Health Care Setting	Role Model for Knowledge Brokering in Healthcare
MFT Medizinischer Fakultätentag der Bundesrepublik Deutschland e. V. (2015) [30]	Germany	Nationaler Kompetenzbasierter Lernzielkatalog Medizin (NKLM)	CanMEDS Framework
Frank et al. (2018) [31]	Canada	CanMEDS 2015 physician competency framework	CanMEDS Framework
Saaristo et al. (2018) [32]	Finland	The comparative and objective measurement of health promotion capacity-building: from conceptual framework to operationalization	The Finnish Framework
Stefl (2008) [33]	United States	Common Competencies for All Healthcare Managers: the Healthcare Leadership Alliance model	The Healthcare Leadership Alliance Model

The models and frameworks that are mentioned most frequently in the included publications are explained below.

The *Core Competencies Framework for Health Promotion (CompHP)* is mentioned in four of the 12 included publications [15, 17, 25, 27]. The model is primarily aimed at health promotion practitioners whose main role and function is health promotion. Ethical values and health promotion knowledge represent the basis at the centre of the further nine health promotion competencies: enable change, mediate through partnership, advocate for health, leadership, communication, needs assessment, planning, implementation, evaluation and research. The combined application of all nine domains, ethical values and health promotion knowledge constitute the CompHP [17].

The *CanMEDS Framework* is mentioned in three [26, 30, 31] of the 12 included publications. It describes the skills that physicians need to respond effectively and competently to the health care needs of their patients. It includes seven roles that a competent physician embraces: medical expert as the integrating role, communicator, collaborator, leader, health advocate, scholar and professional [31].

Glegg and Hoens [29] describe the *Role Model for Knowledge Brokering* in health care. The five role domains in the model are information manager, linking agent, capacity builder, facilitator and evaluator. The model can be used to inform practitioners about knowledge brokering. It can also be used to train professionals and develop assessment strategies [29].

The *Healthcare Leadership Alliance Model* was developed by the Healthcare Leadership Alliance (HLA) through a review and participatory process with practising healthcare managers, who are members of the HLA. The model includes the following competency domains: communication and relationship management, professionalism, leadership, knowledge of the healthcare system and business skills and knowledge [33].

The *Finnish Framework* was assessed and analysed by Saaristo and Hakamäki [32] using empirical data on primary health care in Finland. The framework contains seven competency domains: commitment, management, monitoring and needs assessment, resources, common practices, participation and other core functions [32].

The *Quality Leadership Developmental Competency Model* was developed with the aim of strengthening the skills of health care leaders through self-assessment. It contains 21 competencies grouped in six domains, namely fostering positive change, communication, organisational awareness, self-management, professionalism/professional values and performance improvement over three organisational levels [28].

### Synthesis: role and competency model

**Fig. 2:**
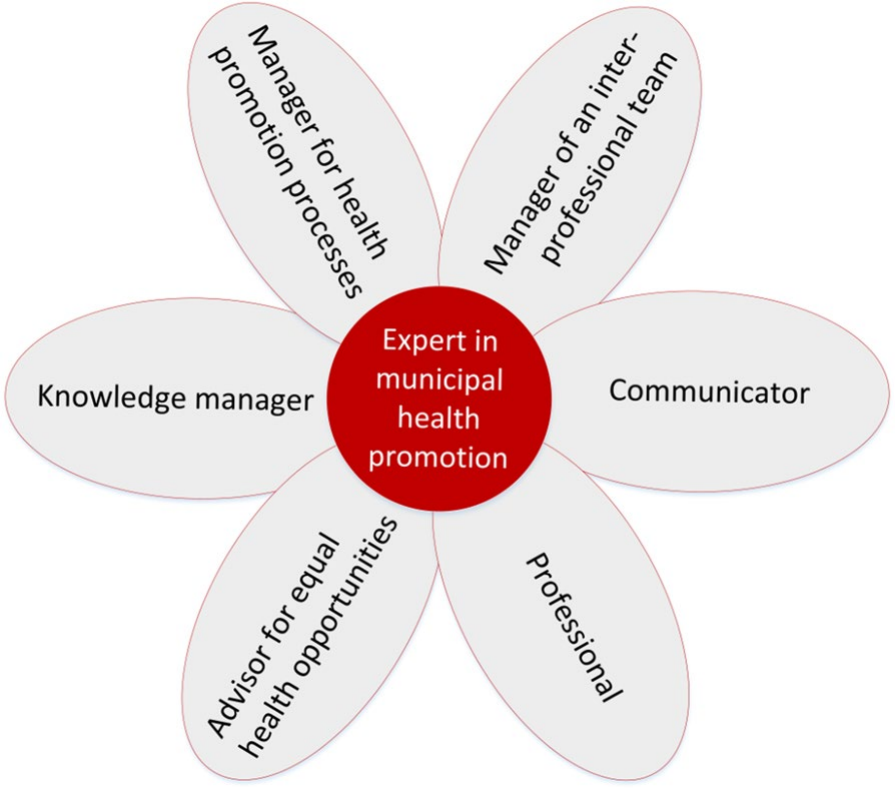
Role and competency model for the coordination of municipal health promotion (own illustration)

As integrative reviews aim to synthesise literature [21], the product of this synthesis of the identified literature is a concept for a model on roles and competencies needed for coordinating municipal health promotion. Especially the CompHP [17], the CanMEDS model [31] and its German translation [30] have influenced the newly developed role and competency model. The other models and frameworks mentioned above have also had an influence on the content. The roles and competencies refer to tasks that a coordinator should perform as an expert in the municipal setting. The role and competency model for the coordination of municipal health promotion is shown in Fig. 2. In particular, the figure shows the roles that a municipal health promotion coordinator must assume. Following the CanMEDS model, the shape of a flower was chosen to illustrate that a key aspect is that the person must be an expert in municipal health promotion. The competencies identified based on the included literature were then mapped to the roles presented in Fig. 2. Table 2 provides a detailed list of the competencies required to fulfil the respective role.

**Table 2:** Competencies for coordinating municipal health promotion

Roles for coordinating municipal health promotion	Competencies
1) Expert in municipal health promotion	Professionalism in municipal health promotion; Integrity; Representation of interests
2) Manager of an interprofessional team	Adaptability; Integration capability; Team skills; Cooperative capacity; Complex thinking; People skills
3) Communicator	Communication skills *(Relationship management skills; Dialogue capability; Language fluency*); Conflict skills *(Problem solving skills*); Appreciation skills; Moderation skills
4) Professional practitioner	Expertise and professional competencies *(Profound expertise in health promotion; Knowledge of health, social and educational policies; Knowledge of the health system; Institutional knowledge; Interprofessional knowledge; Intersectoral knowledge)*; Methodological competencies *(Analytical skills; Scientific working skills; Conceptual skills; Moderation skills; Self-management)*
5) Health equity consultant	Target-specific knowledge; Consultation skills; Identify and advocate for needs and demands of communities/populations; Competency in diversity management; Intercultural competency
6) Knowledge Manager	Lifelong learning; (*Knowledge*) transfer competency; Evaluation competency; Capacity development competency; Enabling competency (*Empowerment*)
7) Manager for health promoting processes	Leadership skills *(Motivational ability)*; Flexibility *(Openness)*; Judgemental ability *(Perseverance; Frustration tolerance)*; Commitment (*Initiative*); Decision-making ability *(Independent work)*; Management competencies *(Planning ability; Organizational skills; Coordination skills)*

In the integrating role as an expert in municipal health promotion, the coordinators should embody integrity and professionally represent the interests of the target group (see Table 2). As managers of an interprofessional team, they require adaptability and the capability to integrate different perspectives and team members. Essential competencies in this role include team skills, cooperative capacity and complex thinking skills, which are crucial for successfully managing interprofessional teams. As communicators in the municipality, they need to be eloquent and possess the ability to deal with conflicts in order to solve problems. Applying these competencies will ensure that all actors stay engaged and informed.

Professional practitioners need to have in-depth knowledge of health promotion and a comprehensive understanding of health, social and educational policies, the health system, as well as institutional, interprofessional and intersectoral knowledge. A professional practitioner combines these expertise and professional competencies with methodological competencies such as analytical, scientific, conceptual, moderation and self-management skills. As health equity consultants, municipal coordinators need to utilize target-specific knowledge and consultation skills to identify and advocate for the needs of their community. They also need to be proficient in diversity management and possess intercultural competency, both of which are vital in advancing health equity. As knowledge managers, they embrace lifelong learning and require competencies in knowledge transfer and evaluation. They should demonstrate strong capacity development and enabling competencies to empower others. In a different role, but at the same time, they are managers of health promotion processes. This requires strong leadership skills including motivational ability, flexibility, commitment and a good decision-making ability, as well as management competencies, such as planning and organisational skills. Further differentiated descriptions of the competencies are provided in the Table 2.

## Discussion

As the structural development and central coordination of municipal health promotion is becoming increasingly important in the context of promoting health equity [5, 11], it is crucial to identify the competencies required to ensure quality and professionalisation. This will contribute to promoting health in municipalities in accordance with the Sustainable Development Goals (SDGs). There is a need for interdisciplinary networking and cooperation between different policy sectors, so that the implementation of measures at the municipal level can be successful at different scales. Various stakeholders from different policy sectors, such as education, environment, transport and business, need to work together. Health should no longer be considered only from a health perspective, but from various intersectoral perspectives and needs to be implemented across many sectors. For this to succeed, it is essential that all stakeholders involved are willing to work together in line with the HiAP approach [4, 5, 7].

In the context of this research, many relevant tasks and necessary competencies for the coordination of municipal health promotion were identified. By synthesising the results according to the integrative review method [21], the researchers developed a role and competency model. The roles in the model are complemented by a comprehensive table (Table 2) showing all identified and required competencies. As the role and competency model focuses on central roles for coordinating municipal health promotion, users should always apply it in combination with the supplementary table to get a comprehensive insight into the required competencies.

This is the first review addressing the topic of roles and competencies of municipal coordination in German- and English-speaking countries for the time period included in the search within an integrative review. This was necessary as there was no overview, e.g. in the form of a review, of this topic at the time.

The integrative review method offers ideal opportunities to achieve the research objective, although it is not widely used and is more commonly applied in the field of health care and nursing science [21]. In contrast to a systematic review, an integrative review allows results to be qualitatively synthesised and presented in an applicable way. This synthesis allows a more holistic understanding of specific findings [22]. To avoid bias, this integrative review followed a very systematic and precise approach to data extraction and data synthesis.

The systematic literature search identified several models and frameworks. The included literature was reviewed and seven roles and 31 competencies for the coordination of municipal health promotion were identified. In order to do justice to the complexity of this field of research, the presentation form of a flower model and a supplementary table was chosen as a synthesis of different models and thematic areas. Nevertheless, it can only claim to be complete within the limitations of the methodology presented. There may be other roles and competencies in practice that could not be found in this integrative review. However, to address this limitation, not only scientific studies but also practice publications were included.

Other limitations of this study include the time lag between the literature search and the publication of the results, and the fact that the review was not pre-registered. It is possible that relevant recent studies (between 2020–2024) were not included, which could affect the validity of our conclusions. Nevertheless, these are fundamental findings that have timeless validity. Despite these limitations, we believe that the roles and competencies identified in this study provide valuable insights and approaches for practice, albeit viewed in the context of the constraints mentioned. The model presented was derived on the basis of scientific publications and represents an abstract, broader perspective on competencies and roles in the sense of a top-down approach. It may not reflect the experiences and perceptions of those involved in coordinating health promotion. Nevertheless, it provides an operationalised and plausible proposal and valuable starting point for further investigation.

Future studies should include a critical evaluation of the model's acceptability, appropriateness, transferability and willingness of health and other professionals to collaborate as part of its practical application as well as its further development. For this purpose, consensus-based, bottom-up approaches should be used with stakeholders from different municipal sectors (e.g. Delphi panels). In a subsequent step, the model will have to be evaluated in its application in practice. Ideally, and after possible adaptations, it can be embedded in university curricula and used to design module guidelines. It can also be transferred and tested in relation to current discussions, such as the establishment of health kiosks in Germany.

## Conclusions

The findings of the present integrative review support a multidisciplinary process by providing information on the competencies that staff should embody in the course of municipal health promotion. These competencies should be transmitted during the education and training process, and they provide guidance for municipalities on the selection of suitable staff and the training of existing staff.

The action model of municipal health promotion by Quilling et al. [8] is a useful supplement to the results presented, as it structures the process of municipal health promotion as well as the corresponding coordination tasks. Both models can be used in combination and provide a suitable, long-term and quality-assured contribution to the structural development of integrated municipal health promotion. The role and competency model can contribute to professionalisation and is applicable in practice.

## Supplementary Information

Additional file 1: Table S1. Detailed summary of findings.

## Data Availability

All data generated or analysed during this study are included in this published article and its supplementary information files (see Table S1).

## Abbreviations

BZgA: Federal Centre for Health Education (German: Bundeszentrale fuer gesundheitliche Aufklaerung)
CompHP: Core Competencies Framework for Health Promotion
HLA: Healthcare Leadership Alliance
PRISMA: Preferred Reporting Items for Systematic reviews and Meta-Analyses
